# The laboratory parameters in predicting the severity and death of COVID-19 patients: Future pandemic readiness strategies

**DOI:** 10.17305/bb.2023.9540

**Published:** 2024-04-01

**Authors:** Ahmad R Alsayed, Syed Imran Ahmed, Anas Osama AL Shweiki, Mustafa Al-Shajlawi, Nancy Hakooz

**Affiliations:** 1 Department of Clinical Pharmacy and Therapeutics, Faculty of Pharmacy, Applied Science Private University, Amman, Jordan; 2 College of Health and Science, School of Pharmacy, University of Lincoln, Lincoln, United Kingdom; 3 School of Pharmacy, The University of Jordan, Amman, Jordan

**Keywords:** Coronavirus disease 2019 (COVID-19), hospitalized patients, laboratory parameters, mortality, predictors, severity

## Abstract

The range of clinical manifestations associated with the infection by the severe acute respiratory syndrome coronavirus 2 (SARS-CoV-2) encompasses a broad spectrum, ranging from flu-like symptoms to the occurrence of multiple organ failure and death. The severity of the coronavirus disease 2019 (COVID-19) is categorized based on clinical presentation and is divided into three distinct levels of severity identified as non-severe, severe, and critical. Although individuals of all age groups are susceptible to SARS-CoV-2 infection, middle-aged and older adults are more frequently impacted, with the latter being more likely to develop severe illness. Various laboratory characteristics observed in hospitalized COVID-19 patients have been correlated with adverse outcomes. These include elevated levels of D-dimer, liver enzymes, lactate dehydrogenase (LDH), C-reactive protein (CRP), ferritin, prothrombin time (PT), and troponin, as well as decreased lymphocyte and platelets counts. This review investigated the relationship between baseline clinical characteristics, initial laboratory parameters upon hospital admission, and the severity of illness and mortality rates among COVID-19 patients. Although the COVID-19 pandemic has concluded, understanding the laboratory predictors of the disease severity and mortality remains crucial, and examining these predictors can have long-term effects. Such insights can help healthcare systems manage resources more effectively and deliver timely and appropriate care by identifying and targeting high-risk individuals. This knowledge can also help us better prepare for future pandemics. By examining these predictors, we can take steps to protect public health and mitigate the impact of future pandemics.

## Introduction

The initial cases of the infectious coronavirus disease 2019 (COVID-19) were documented in December 2019 in Wuhan, a city in the Hubei Province of China. Subsequently, this city emerged as the focal point of the ensuing global outbreak. The etiology of COVID-19 is attributed to a viral pathogen known as the severe acute respiratory syndrome coronavirus 2 (SARS-CoV-2) [1].

COVID-19 is an infectious disease caused by the SARS-CoV-2 virus [1]. SARS-CoV-2 is a positive-stranded RNA virus that is enveloped. It is a beta coronavirus, belonging to the same subgenus as the severe acute respiratory syndrome coronavirus (SARS-CoV), as well as other bat coronaviruses, but it exists in a distinct clade [1]. The virus generally causes mild to moderate respiratory illness in most infected individuals, who typically recover without requiring special treatment [2]. However, some patients develop life-threatening illnesses that require medical intervention [3].

The clinical presentation of SARS-CoV-2 infection varies widely, ranging from symptoms resembling influenza to severe manifestations such as multi-organ dysfunction leading to mortality [4]. The severity of COVID-19 is categorized based on the clinical presentation, and can be categorized into three distinct levels of severity identified as non-severe, severe, and critical [4, 5]. Individuals afflicted with critical illness often exhibit certain distinguishing features, including the manifestation of acute respiratory distress syndrome (ARDS), sepsis, septic shock, and organ dysfunction, or the need for vasopressor therapy. Those with severe illness exhibit symptoms of pneumonia and meet at least one of the following criteria: peripheral capillary oxygen saturation (SpO_2_) below 90% while breathing ambient air, a respiratory rate exceeding 30 breaths per minute, or the experience of severe respiratory distress. Non-severe patients can be identified by the absence of indicators that would classify them as either severe or critical cases [4–6].

Individuals across all age groups are susceptible to SARS-CoV-2 infection. However, the middle-aged and older adult demographics are more frequently impacted, with older adults exhibiting a higher propensity for severe symptom manifestation associated with the disease. The median age of hospitalized patients with confirmed COVID-19 ranged from 49 to 56 years across multiple cohorts [3, 7, 8].

The laboratory characteristics of patients admitted to hospitals for COVID-19 have also been correlated with unfavorable outcomes. The observed laboratory findings encompassed elevated levels of D-dimer, liver enzymes, lactate dehydrogenase (LDH), C-reactive protein (CRP), ferritin, prothrombin time (PT), and troponin. Conversely, decreased levels of lymphocyte and platelet counts were also noted [4, 6, 9–11]. A study conducted in China involving 138 hospitalized patients revealed that individuals who did not survive exhibited a progressive decrease in lymphocyte count and an increase in D-dimer levels over time. In contrast, survivors maintained relatively consistent levels of these indicators [7].

Several risk factors have been identified as contributing to the progression of COVID-19 into more severe and critical categories. These factors include advanced age, male gender, and underlying comorbidities, such as obesity, hypertension, diabetes, lung, heart, liver, and kidney diseases, immunodeficiency, and malignancies. Furthermore, socioeconomic status, diet, lifestyle, geographical location, ethnicity, exposed viral load, timing of treatment initiation, and healthcare quality have all been shown to influence individual outcomes. Studies indicate that elevated levels of CRP and radiographic evidence upon admission are correlated with COVID-19 pneumonia, entry into the intensive care unit (ICU), and mortality rates [6, 12–16].

This review examined the relationship between baseline clinical features, initial laboratory data upon hospital admission, and both the disease severity and fatality rates in individuals diagnosed with COVID-19. Even though the COVID-19 pandemic has concluded, the significance of identifying laboratory predictors for virus severity and fatality rates remains pertinent. Despite the crisis being resolved, analyzing these predictors has enduring implications. By identifying and prioritizing individuals at high risk, healthcare systems can optimize resource allocation and provide timely and appropriate care. Such understanding also enhances our preparedness for potential future pandemics. The mitigation of public health risks and the prevention of future catastrophic events can be achieved by examining laboratory predictors for COVID-19 severity and mortality.

## Epidemiology of COVID-19

In December 2019, Wuhan became the epicenter of the initial outbreak of COVID-19 cases. Seroprevalence surveys conducted in Europe and the United States suggest that the reported COVID-19 cases underestimate the overall disease burden. After accounting for potential false positives and negatives, these surveys suggest that the actual incidence of COVID-19 cases is estimated to be at least ten times higher than the registered cases, as indicated by seropositivity rates [17, 18].

The global count of confirmed COVID-19 cases has surpassed 500 million. A comprehensive interactive map displaying the worldwide distribution of confirmed cases as of March 2023 is available at the following link: https://coronavirus.jhu.edu/map.html.

A research study that utilized multiple data sources, such as databases with information on case numbers, COVID-19-related fatalities, and seroprevalence, estimated that as of November 2021, more than three billion people, accounting for approximately 44% of the global population, had experienced at least one SARS-CoV-2 infection [19]. It is estimated that over 33% of these total cases occurred in the South Asian region, including India.

## Pathophysiology of COVID-19

The COVID-19 pandemic has spurred extensive research into its pathophysiology. Understanding the underlying mechanisms is crucial for the development of effective treatments and management strategies.

SARS-CoV-2 primarily enters host cells by binding to the angiotensin-converting enzyme 2 (ACE2) receptor. The viral spike (S) protein binds to the ACE2, facilitating membrane fusion and the release of viral RNA into the host cell. This interaction triggers a series of downstream events that include viral replication and the initiation of host immune responses [20].

The SARS-CoV-2 virus is structurally composed of four proteins: the S protein, membrane (M) protein, enveloped (E) protein, and nucleocapsid (N) protein. The N protein houses the RNA genome of the virus, while the other three proteins form the viral envelope. The S protein consists of two functional subunits: S1, which binds to the ACE2 receptor, and S2, which mediates viral fusion with the host cell membrane [21, 22]. Once inside the host cell, the viral RNA takes over the host cell’s replication machinery to initiate viral genome replication, polypeptide chain synthesis, and the formation of the replication–transcription complex (RCT). This complex is required for the synthesis of sub-genomic RNAs and structural proteins (E and N proteins). The viral envelope is critical for viral assembly, release, and pathogenicity [21, 22].

The immune response to SARS-CoV-2 involves a complex interplay between the innate and adaptive arms of the immune system. Various immune cells, such as macrophages, dendritic cells, and T lymphocytes, are activated. Dysregulation of these immune responses can lead to a cytokine storm, which is characterized by the excessive production of pro-inflammatory cytokines [23, 24].

Many cases of SARS-CoV-2 infection are often accompanied by an overwhelming cytokine storm, marked by elevated levels of interleukin 6 (IL-6), IL-1β, and tumor necrosis factor-alpha (TNF-α). This cytokine dysregulation contributes to tissue damage, vascular leakage, and systemic inflammation [25].

COVID-19 can also result in ARDS, which is characterized by diffuse alveolar damage, hyaline membrane formation, and impaired gas exchange. Both the virus-induced cytokine storm and direct viral damage to lung tissue are pivotal factors in the development of ARDS [26].

Patients with severe COVID-19 are prone to coagulation abnormalities, including disseminated intravascular coagulation (DIC). Factors, such as endothelial dysfunction, platelet activation, and increased von Willebrand factor (VWF) levels contribute to the hypercoagulable state, increasing the risk of thrombotic events [27, 28].

Although primarily a respiratory illness, COVID-19 is associated with extra-pulmonary manifestations. These include cardiovascular complications, renal dysfunction, gastrointestinal symptoms, and neurological effects, suggesting a systemic impact of the virus [29–31].

Some individuals experience symptoms that persist beyond the acute phase of the infection, commonly referred to as “long COVID”. Symptoms, such as fatigue, cognitive impairment, and respiratory issues may result from ongoing inflammation and immune dysregulation [32].

The pathophysiology of COVID-19 is a dynamic and intricate process involving viral entry, immune responses, inflammation, and multi-organ involvement. While our understanding of the disease has significantly advanced, ongoing research is imperative for uncovering the complete spectrum of mechanisms driving its pathogenesis.

## Risk factors for COVID-19-associated hospitalization

SARS-CoV2 has the ability to infect individuals across all age groups, although it is more frequently observed to affect individuals in middle-aged and older demographics. Furthermore, older adults are also more likely to experience severe illnesses. Multiple studies conducted on hospitalized COVID-19 patients have revealed a median age ranging between 49 and 56 years [3, 7, 8]. Additionally, older age has been associated with increased mortality rates [2, 6, 33, 34].

Severe illness, characterized by hospitalization, admission to the ICU, or death, has been associated with multiple comorbidities and underlying conditions. One study involving 5416 patients showed that hospitalization rates were higher among those with three or more underlying conditions. These conditions include obesity (body mass index [BMI] ≥ 30 kg/m^2^) and severe obesity (BMI ≥ 40 kg/m^2^), chronic kidney disease, diabetes, hypertension, and asthma [35]. Similarly, another study found that risk factors for hospitalization included heart failure, male sex, chronic kidney disease, and any elevated BMI levels (e.g., BMI > 40) [36].

Certain demographic characteristics have also been correlated with more severe COVID-19 illnesses. Several cohort studies have indicated that males experience a higher number of critical cases and deaths [33, 34, 37–39].

## Clinical presentation of COVID-19

### Signs and symptoms of COVID-19

Symptomatic COVID-19 patients typically present with respiratory syndrome, with fever and cough being commonly recorded [40, 41]. According to a study conducted in Wuhan, China, involving 99 patients, the most prevalent reported symptoms were fever (83%), cough (82%), shortness of breath (31%), muscle ache (11%), confusion (9%), and headache (8%). Other symptoms, such as rhinorrhea, vomiting, diarrhea, nausea, sore throat, and chest pain, were less common, occurring in fewer than 5% of cases. Based on imaging examinations, 75% of patients exhibited bilateral pneumonia, 14% displayed multiple mottling and ground-glass opacity, and 1% had pneumothorax [8]. Similarly, other studies have reported that fever, fatigue, and dry cough were the most common symptoms [7, 42].

In another study that included 90 COVID-19 patients, the most common symptoms were cough (81%), headache (76%), fever (either subjective or measured at ≥ 38.0 ^∘^C [≥ 100.4 ∘F]) (64%), loss of taste and/or smell (62%), and nasal congestion (62%) [43].

## Diagnosis of COVID-19

COVID-19 cannot be consistently distinguished from other viral respiratory infections solely based on specific clinical features [44]. Some studies have suggested that loss of smell or taste is the most significantly associated symptom with a positive SARS-CoV-2 test. Additionally, dyspnea is another symptom suggestive of COVID-19 that often develops after several days of the illness [43, 45–47].

Nucleic acid amplification testing (NAAT) is a diagnostic method used to identify SARS-CoV-2 infection by detecting the virus’ genetic material. Various techniques, such as reverse transcription-polymerase chain reaction (RT-PCR) and isothermal amplification, can be used in NAATs to amplify and identify the virus. The preferred initial diagnostic test for COVID-19 is a NAAT, most commonly RT-PCR utilized to identify the virus RNA from upper respiratory tract specimens [48–53]. Around the world, various RT-PCR assays are in use, and each amplifies and detects a distinct portion of the SARS-CoV-2 genome. Some approaches target several genes, including the S protein, N protein, and E protein genes, as well as specific regions within the first open reading frame like the RNA-dependent RNA polymerase (*RdRp*) gene [54]. In comparison, rapid RT-PCR assays demonstrate performance levels similar to conventional laboratory-based NAATs, although potentially exhibiting lower sensitivity [55].

There is a lack of systematic evaluation concerning the accuracy and predictive utility of SARS-CoV-2 NAATs, despite them being highly specific tests [56, 57]. While NAATs exhibit high analytic sensitivity under ideal conditions, their clinical performance is more variable [58, 59]. Estimates for false-negative rates vary widely, ranging from less than 5% to as high as 40%. These estimates, however, are constrained by the absence of a universally accepted reference standard for comparison [53, 59, 60].

Antigen tests for SARS-CoV-2 offer the advantage of quick implementation and point-of-care utility, making them potentially more accessible and faster than some NAATs. Various at-home antigen tests allow individuals to test themselves without needing to be presented to a medical care or testing site. However, antigen tests generally exhibit lower sensitivity compared to the NAATs [61–63].

Serologic assays aim to detect SARS-CoV-2 antibodies in the blood and can identify individuals who have previously been infected with the virus and those who have been infected three to four weeks prior to the testing. However, due to their limited reactivity during the initial days to weeks of infection, serologic tests demonstrate minimal diagnostic utility in the acute setting [64, 65].

## Laboratory findings of COVID-19 patients

Several studies have reported abnormalities of the laboratory parameters in hospitalized COVID-19 patients (Table 1). Notably, these abnormalities appear to be significantly higher in patients with more severe forms of the disease, as reflected in Table 1.

**Table 1:** Laboratory features associated with severe COVID-19

**Parameter**	**Possible threshold**	**Evidence**	**References**
D-dimer	> 1 mg/L (normal range: < 0.5 mg/L)	ICU patients had a higher level of D-dimer than non-ICU patients. In addition, the level of D-dimer continued to increase until death occurred.	[3, 7]
		The number of patients with D-dimer ≥ 0.5 mg/L was higher in severe cases than in non-severe.	[78]
		Non-survivor patients had a higher level of D-dimer than survivor patients. In addition, the multivariate logistic regression model showed that D-dimer greater than 1 mg/L on admission was associated with higher mortality.	[9]
		A higher level of D-dimer was a risk factor for developing ARDS and progression from ARDS to death.	[11]
		Critical patients had a higher level of D-dimer than moderate and severe patients. In addition, the multivariable logistic regression model showed that increased D-dimer (> 2 mg/L; OR ═ 4.41 [95% CI 1.06–18.30]; *P* ═ 0.041) was associated with an increased risk of death.	[4]
		Death during hospitalization was higher among patients with D-dimer ≥ 2.0 mg/L than those with D-dimer < 2.0 mg/L on admission.	[79]
		Out of all the cases with a D-dimer level of > 1 mg/L, 86.7% were non-survivors, 63.3% had severe disease, and 86.3% had critical disease (*P* < 0.001).	[6]
CRP	>100 mg/L (normal range: < 8.0 mg/L)	Severe patients had a higher level of CRP than non-severe.	[4, 78]
		There was a significant difference in CRP levels between the deceased and discharged groups.	[80]
		COVID-19 patients with cardiac injury had a higher CRP level than patients without cardiac injury.	[10]
		Patients with D-dimer levels ≥ 2.0 mg/L had a higher CRP level (*P* < 0.001).	[79]
		IL-6 and CRP were highly predictive of the need for invasive ventilation.	[81]
		Out of all the cases with a CRP level of > 100 mg/L, 79.0% were non-survivors, 67.9% had severe disease, and 75.5% had critical disease (*P* < 0.001).	[6]
PT	NA	On admission, the PT level was higher in ICU patients than in non-ICU patients (*P* ═ 0.012).	[3]
		The level of PT was higher in non-survivor patients than in survivor patients (*P* ═ 0.0004). In addition, the odds of in-hospital death showed that elevated PT was associated with death.	[9]
		PT was associated with ARDS development but not associated with progression from ARDS to death.	[11]
		PT was significantly higher in critically ill patients than in those with moderate and severe disease. Furthermore, PT (> 16 s; OR ═ 4.94 [95% CI 1.50–16.25]; *P* ═ 0.0094) was associated with an increased risk of death.	[4]
		Patients with D-dimer levels ≥ 2.0 mg/L had a higher level of PT (*P* < 0.001).	[79]
LDH	> 245 U/L (normal range: 110 to 210 U/L)	LDH level was higher in non-survivor than survivor patients. In addition, the elevated LDH was associated with death.	[9]
		Among 198 patients, the median (IQR) value of LDH was 307.50 U/L (232.25–389.25). Furthermore, higher LDH was a risk factor related to the development of ARDS and progression from ARDS to death.	[11]
		LDH values were significantly different between moderate, severe, and critical patients (*P* < 0.001).	[4]
		Heart rate, markers of inflammation, and LDH level at admission were significantly associated with respiratory failure.	[81]
Troponin	> 2× the upper limit of normal (normal range for high-sensitivity troponin T: females 0–9 ng/L; males 0–14 ng/L)	Non-survivor patients had a higher level of high-sensitivity cardiac troponin I than survivors. In addition, the elevated high-sensitivity cardiac troponin I was associated with death.	[9]
		The level of hypersensitive troponin I was higher in ICU patients than in non-ICU patients	[7]
		Cardiac troponin levels were significantly different between deceased and discharged patients.	[80]
		Patients with cardiac injury had higher levels of high-sensitivity troponin I than those without cardiac injury.	[10]
Ferritin	> 500 mcg/L (normal range: females 10–200 mcg/L; males 30–300 mcg/L)	Levels of serum ferritin were higher in non-survivor patients than in survivor patients. Odds of in-hospital death showed that elevated ferritin was associated with death.	[9]
		Serum ferritin was one of the several factors associated with ARDS development but not associated with death.	[11]
		Serum ferritin values significantly differed between moderate, severe, and critical patients (*P* <0.05).	[4]
		Out of all the cases with a ferritin level of > 500 mcg/L, 25.0% had severe disease, and 83.3% had critical disease (*P* < 0.001).	[6]
WBC count	NA	WBC count was higher in ICU patients than in non-ICU patients. Also, it was higher in non-survivor than in the survivor patients.	[7]
		On admission, the blood counts of patients showed leucopenia (WBC count less than 4 × 10^9^/L; 10/40 [25%] patients).	[3]
		WBC levels were significantly different between deceased and discharged patients.	[80]
		WBC level was higher in non-survivor than in the survivor patients.	[9]
		Patients with cardiac injury had a higher level of WBC than patients without cardiac injury.	[10]
		Out of all the cases with a WBC count of > 10 × 10^3^/µL, 64.6% were non-survivors, 42.9% had severe disease, and 61.5% had critical disease (*P* < 0.001).	[6]
Neutrophil count	NA	The neutrophil count was higher in ICU than in non-ICU patients. In addition, the neutrophil count continued to increase until death occurred in deceased patients.	[7]
		68 of 197 (34.5%) patients had neutrophilia, with a median (IQR) value of 4.47 × 10^9^/mL (2.32–7.70). Furthermore, neutrophilia was a risk factor related to ARDS development and progression from ARDS to death.	[11]
		Patients with D-dimer levels ≥ 2.0 mg/L had a higher neutrophil count (*P* < 0.001).	[79]
		Out of all the cases with a neutrophil count of > 7 × 10^9^/L, 81.3% were non-survivors, 55.0% had severe disease, and 76.5% had critical disease (*P* < 0.001).	[6]
ALT	NA	On admission, laboratory findings of 1099 patients showed that ALT levels were less frequently elevated. The no./total (%) of patients who had ALT > 40 U/L were 38/135 (28.1%) in severe patients and 120/606 (19.8%) in non-severe patients.	[78]
		ALT levels were higher in ICU than in non-ICU patients.	[7]
		ALT levels were higher in non-survivor than survivor patients, and the elevated ALT level was associated with death.	[9]
		A laboratory finding of 198 patients showed that 21.7% exhibited liver injury with elevated ALT. The median (IQR) value of ALT among patients with liver injury was 31.00 U/L (19.75–47.00).	[11]
		Out of all the cases with an ALT level of > 53 IU/L, 35.5% had severe disease, and 29.1% had critical disease (*P* < 0.001).	[6]
AST	NA	AST levels were higher in ICU than in non-ICU patients.	[7]
		On admission, AST levels were less frequently elevated. Furthermore, 56/142 (39.4%) patients who had AST > 40 U/L were in the severe category, and 112/615 (18.2%) were in the non-severe category.	[78]
		29.8% of patients exhibited liver injury with elevated AST. The median (IQR) value of AST in U/L was 33.00 (26.00–45.00). In addition, AST was one of the several factors associated with ARDS development but not associated with progression from ARDS to death.	[11]
		AST levels were higher in patients with cardiac injury than in patients without cardiac injury.	[10]
		Out of all the cases with an AST level of > 47 IU/L, 60.5% were non-survivors, 44.5% had severe disease, and 54.4% had critical disease (*P* <0.001).	[6]
CK	NA	CK–MB levels were higher in ICU patients than in non-ICU patients.	[7]
		CK levels were higher in non-survivor than in survivor patients. Furthermore, the odds of hospital death showed that CK was one of several factors associated with death.	[9]
		CK–MB levels were higher in patients with cardiac injury than in those without cardiac injury.	[10]
Creatinine	NA	ICU patients had a higher level of creatinine than non-ICU patients. In addition, the level of creatinine continued to increase until death occurred.	[7]
		The no./total (%) of patients who had creatinine ≥ 133 µmol/L were 6/138 (4.3%) in severe patients and 6/614 (1.0%) in non-severe patients.	[78]
		There was a significant difference in blood creatinine levels between the deceased and discharged patients.	[80]
		Odds of in-hospital death showed that creatinine was one of several factors associated with death.	[9]
		Creatinine was one of several factors associated with ARDS development but not associated with progression from ARDS to death.	[11]
		The median (IQR) of creatinine during hospitalization was 11.5 mg/L (7.2–19.2) for patients with cardiac injury and 6.4 mg/L (5.4–7.8) for patients without cardiac injury.	[10]
		A study conducted to evaluate predictors of respiratory failure showed that creatinine levels at admission were significantly associated with respiratory failure.	[81]
		Out of all the cases with creatinine levels of > 104 µmol/L, 40.2% were non-survivors (*P* ═ 0.001), 20.2% had severe disease, and 38.5% had critical disease (*P* ═ 0.004).	[6]
Procalcitonin	NA	According to a study conducted on 138 patients (36 ICU and 102 non-ICU patients), no (%) of patients who had procalcitonin levels of ≥ 0.05 ng/mL were 27 (75.0%) from the ICU group and 22 (21.6%) from the non-ICU group.	[7]
		A study that included 1099 patients (173 severe and 926 non-severe patients) showed that no./total (%) of patients who had procalcitonin levels of ≥ 0.5 ng/mL were 16/117 (13.7%) in severe patients and 19/516 (3.7%) in non-severe patients.	[78]
		According to a study on 41 patients (13 ICU and 28 non-ICU patients), four ICU patients developed secondary infections. Three of the four patients with secondary infection had procalcitonin greater than 0.5 ng/mL (0.69 ng/mL, 1.46 ng/mL, and 6.48 ng/mL).	[3]
		Odds of in-hospital death showed that procalcitonin was associated with death.	[9]
		According to the study which included 416 patients (82 with cardiac injury and 334 without cardiac injury), the median (IQR) of procalcitonin among patients with cardiac injury was 0.27 ng/mL (0.10–1.22) and 0.06 ng/mL (0.03–0.10) among patients without cardiac injury.	[10]
BUN	NA	BUN levels were higher in ICU patients than in non-ICU.	[7]
		BUN levels were significantly different between deceased and discharged patients.	[80]
		Out of all the cases with BUN levels of > 8.9 mmol/L, 78.0% were non-survivors, 39.4% had severe disease, and 73.1% had critical disease (*P* < 0.001).	[6]
Absolute lymphocyte count	< 0.8 × 10^9^/L (normal range for age ≥ 21 years: 1.8 to 7.7 × 10^9^/L)	ICU patients had a lower lymphocyte count than non-ICU patients. In addition, the level of lymphocyte counts continued to decrease until death occurred.	[7]
		According to a study conducted on 41 patients, the blood count on admission showed that 26 patients had lymphocytopenia (lymphocyte count < 1.0 × 10^9^/L).	[3]
		Lymphocytopenia was more prominent among patients with severe diseases than those with the non-severe disease.	[78]
		There was a significant difference in absolute lymphocyte counts between the deceased and discharged groups.	[80]
		Patients with lymphocytopenia showed a higher odd of in-hospital death.	[9]
		Laboratory findings of 194 patients showed that 64.0% of patients had lymphocytopenia (value of lymphocytes were 0.91 × 10^9^/mL (IQR 0.60–1.29). In addition, lymphocyte counts were one of several factors associated with the development of ARDS but not associated with progression from ARDS to death.	[11]
		A comparison between cardiac injury patients and non-cardiac injury patients showed a lower median lymphocyte count (median [IQR]; 600 cells/µL [400–900] vs 1000 cells/µL [800–1400]), with significant differences.	[10]
		Increased neutrophil-to-lymphocyte ratio (≥ 9.13; OR ═ 5.39 [95% CI 1.70–17.13]; *P* ═ 0.0042) was associated with an increased risk of death.	[4]
		Patients with D-dimer levels ≥ 2.0 µg/mL had a lower lymphocyte count (*P* < 0.001).	[79]
		Out of all the cases with a lymphocyte count of < 0.8 × 10^9^/L, 78.0% were non-survivors, 73.4% had severe disease, and 76.0% had critical disease (*P* < 0.001).	[6]
Platelet count	NA	The median (IQR) of platelet count per mm^3^ in severe patients was 137,500 (99,000–179,500) and in non-severe patients 172,000 (139,000–212,000). In addition, thrombocytopenia was present in 36% of the patients.	[78]
		There was a significant difference in platelet levels between the deceased and discharged groups.	[80]
		Non-survivor patients had lower platelet counts than survivor patients (*P* < 0.0001).	[9]
		A comparison between cardiac injury patients and non-cardiac injury patients showed a lower median of platelet count (median [IQR]; 172 cells × 10^3^/µL [111–215] vs 216 cells × 10^3^/µL [165–273]), with significant differences.	[10]
		Counts of eosinophils and platelets were significantly lower in patients with critical disease than in those with severe disease (*P* < 0.0001). In addition, thrombocytopenia (platelet count < 100 × 10^9^/L) was recorded in 42 (49%) out of 86 patients with the critical disease, which is a significantly higher frequency than in patients with severe (20 [14%] out of 145) and moderate (9 [6%] out of 149) disease (*P* < 0.0001 for both). Furthermore, thrombocytopenia (platelet count < 100 × 10^9^/L; OR ═ 8.33 [95% CI 2.56–27.15]; *P* ═ 0.00045), was associated with an increased risk of death.	[4]
		Patients with D-dimer levels ≥ 2.0 mg/L had lower hemoglobin (*P* ═ 0.003) and platelet count (*P* ═ 0.009).	[79]
Hemoglobin	NA	Patients with critical disease had a lower level of hemoglobin than patients with severe or moderate disease.	[82]
		No significant difference in hemoglobin levels was observed between survivor and non-survivor patients.	[9]
Albumin	NA	Non-survivor patients had a lower level of albumin than survivor patients.	[9]
		There was a difference in albumin levels among patients with critical, severe, moderate, and mild disease. Furthermore, multinomial logistic regression analyses showed that abnormal albumin levels correlated with disease severity.	[82, 83]
		Out of all the cases with an albumin level of < 3.5 g/dL, 91.8% were non-survivors, 64.7% had severe disease, and 90.2% had critical disease (*P* < 0.001).	[6]

Several studies’ results reporting abnormalities of the laboratory parameters in hospitalized COVID-19 patients have been shown in the table. COVID-19: Coronavirus disease 2019; ICU: Intensive care unit; ARDS: Acute respiratory distress syndrome; CRP: C-reactive protein; IL-6: Interleukin 6; PT: Prothrombin time; LDH: Lactate dehydrogenase; WBC: White blood cell; ALT: Alanine transaminase; AST: Aspartate aminotransferase: CK: Creatine kinase; CK–MB: Creatine kinase–myocardial band; BUN: Blood urea nitrogen; NA: Not applicable.

A recent retrospective study conducted in Jordan aimed to investigate the correlation between the initial laboratory parameters of hospitalized COVID-19 patients and the incidence of severity and mortality [6]. A significant statistical correlation was observed between exceeding certain laboratory parameters’ cut-off values and the severity or mortality of the condition. These parameters, outlined in Table 1, when surpassed, were found to significantly elevate the risk of mortality. The study reported the highest odds ratio for individuals with albumin levels falling below 3.5 g/dL (OR ═ 14.318; *P* < 0.001) (Table 1) [6]. Furthermore, oxygen saturation upon hospital admission stood out as the most potent predictor of mortality among COVID-19 patients aged 18–50, according to another recent study [66].

The most common laboratory abnormalities seen in hospitalized COVID-19 patients include lymphocytopenia accompanied by an elevated neutrophil count, thrombocytopenia, anemia, raised LDH levels, D-dimer, CRP, liver enzymes, and decreased albumin levels [67–69]. Specific markers, such as lymphocytopenia, neutropenia, elevated serum liver enzymes, LDH, CRP, and ferritin, have been associated with an elevated risk of severe disease [66, 70, 71]. Elevated levels of CRP and reduced albumin levels are crucial indicators of severe disease progression [72, 73], indicating the development of cytokine storm in COVID-19 patients.

The cut-off values for these laboratory parameters can serve as reliable indicators for assessing the risk of severity and mortality in COVID-19 patients, thereby providing vital information that could be instrumental in tailoring medical care.

### Antibody titers

In early 2020, the emergence of the novel coronavirus, SARS-CoV-2, and the subsequent global spread of COVID-19 prompted extensive research into various aspects of the virus’s behavior, including the measurement of antibody responses. Studies during this period shed light on the relationship between antibody levels and disease outcomes. Likewise, previous research on the SARS-CoV virus also offered insights into the correlation between antibody response levels and disease severity.

Early literature on SARS-CoV-2 indicated that individuals who experienced severe cases of COVID-19 tended to exhibit higher antibody titers. Studies such as those by Long et al. [74] and Wu et al. [75] observed that patients with more severe symptoms had elevated levels of neutralizing antibodies compared to those with milder symptoms. These findings suggested that a robust immune response, as reflected by higher antibody titers, might be associated with a more intense inflammatory response, potentially contributing to the severity of the disease. It was also hypothesized that high antibody levels could lead to immune dysregulation, promoting cytokine storms, and subsequent organ damage.

Comparatively, earlier research on the original SARS-CoV virus, responsible for the SARS outbreak in 2002–2003, also highlighted the connection between antibody responses and disease severity. In studies like that by Liu et al. [76], it was observed that patients who survived SARS tended to have higher antibody titers against the virus. This implied that a robust immune response played a role in overcoming the infection and preventing disease progression. However, it is worth noting that there were also instances where excessively high antibody titers led to immune-mediated pathology, as observed in animal models.

While early literature pointed toward a correlation between high antibody titers and disease severity for both SARS-CoV-2 and the original SARS-CoV virus, further research has nuanced this understanding. More recent studies have revealed that the relationship between antibody levels and disease outcomes is complex, influenced by factors, such as the timing of antibody development, the types of antibodies produced, and the interplay between the immune response and the virus itself [77]. As the pandemic has progressed, it has become increasingly clear that other immune responses, such as cellular immunity, also play pivotal roles in determining disease severity and long-term protection.

In conclusion, the early literature surrounding SARS-CoV-2 and the original SARS-CoV virus indicated a correlation between high antibody titers and disease severity. However, subsequent research has illuminated the intricate and multifaceted nature of the immune response to these viruses. It is important to consider not only antibody levels but also other aspects of immunity to gain a comprehensive understanding of disease progression and immunity development.

### D-dimer

A retrospective study conducted in Wuhan, China, included 138 hospitalized patients with novel coronavirus-infected pneumonia (NCIP) [7]. According to this study, the level of D-dimer was higher in ICU patients than in non-ICU patients, with a median (IQR) of 414 mg/L (191–1324) compared to 166 mg/L (101–285), respectively. After tacking the dynamic profiles of 33 patients, of whom 5 deceased and 28 survived, the D-dimer levels continued to increase until death occurred [7]. Similarly, another study based in the same city, involving 41 confirmed COVID-19 patients, also reported higher D-dimer levels in ICU patients compared to non-ICU patients, with a median (IQR) of 2.4 mg/L (0.6–14.4) against 0.5 mg/L (0.3–0.8), respectively, *P* ═ 0.0042 [3].

Another study in Wuhan, China, that included 191 hospitalized COVID-19 cases found that the D-dimer values were higher in the 54 non-survivor cases compared to the 137 survivors, with a median (IQR) of 5.2 mg/L (1.5–21.1) vs 0.6 mg/L (0.3–1.0). Furthermore, multivariable logistic regression model findings indicated that D-dimer levels higher than 1 mg/L were associated with increased odds of death [9]. Another study of 201 patients in Wuhan revealed that a higher level of D-dimer was a risk factor for developing ARDS and for progression from ARDS to death [11].

Liao et al. [4] analyzed 380 hospitalized COVID-19 patients and found that the D-dimer levels were higher in critical patients than in severe and moderate patients. A multivariable logistic regression model analysis further revealed that increased D-dimer levels (> 2 mg/L; OR ═ 4.41 [1.06–18.30]; *P* ═ 0.041) were associated with a higher risk of death. Moreover, the D-dimer levels differed significantly between the survivor and non-survivor groups [4]. Contrarily, another study conducted in China, involving 1099 patients, showed that elevated D-dimer levels were less common upon admission [78].

In a separate retrospective study in Wuhan, 267 out of 343 (77.8%) hospitalized patients exhibited D-dimer levels < 2 mg/L, while 67 patients had D-dimer levels > 2 mg/L [79]. A significant difference in mortality rates was observed between the two groups, with a higher incidence of death among patients with D-dimer levels > 2 mg/L. The study showed that D-dimer levels ≥ 2.0 mg/L were significant predictors of subsequent deaths (*P* < 0.001; HR ═ 51.5, 95% CI 12.9–206.7). Furthermore, patients with D-dimer levels ≥ 2.0 mg/L also had statistically significant lower lymphocyte, hemoglobin, and platelet counts and higher levels of neutrophils, CRP, and PT [79].

### C-reactive protein (CRP)

A retrospective study conducted in China analyzed 150 laboratory-confirmed COVID-19 patients, comprising 68 death cases and 82 discharged cases. The study revealed a significant difference in CRP levels between the two groups (*P* < 0.001), with higher CRP levels found in death cases [80]. Another study based in Wuhan, China, which included 1099 patients, showed elevated CRP levels in the majority of the participants. Moreover, 110 out of 135 (81.5%) patients with CRP levels ≥ 10 mg/L were classified as severe cases, whereas 371 out of 658 (56.4%) fell into the non-severe category [78].

An additional cohort study involving 416 patients, conducted in Wuhan, China, found elevated CRP levels, with a median (IQR) of 45 mg/L (14–85). Additionally, a comparison was made between patients with cardiac injury to those without cardiac injury. The findings revealed that the median CRP levels were higher in patients with cardiac injury (median [IQR] of 102 mg/L [64–170]) compared to patients without cardiac injury (median [IQR] of 37 mg/dL [10–73]) [10]. Yet another study in the same city, involving 380 patients, stratified and compared the participants based on disease severity. The study found significant differences in median CRP levels among moderate, severe, and critical cases, which were 10.15, 40.6, and 92.79 mg/L, respectively [4].

According to a previous study involving 343 patients, patients with a D-dimer level of ≥ 2.0 mg/L exhibited significantly higher CRP levels (*P* < 0.001) [79]. Further supporting the significance of CRP levels, Herold et al. [81] found that IL-6 and CRP were strong predictors of the necessity for invasive ventilation.

### Prothrombin time (PT)

Previous research conducted in China, involving 41 hospitalized patients, reported that PT was higher in ICU patients compared to non-ICU patients, with a median (IQR) of 12.2 s (11.2–13.4) and 10.7 s [9.8–12.1], respectively (*P* ═ 0.012) [3]. Another study aimed at describing the outcomes and clinical characteristics of COVID-19 patients with ARDS found that elevated PT was associated with the development of ARDS [11].

A study by Zhou et al. observed a variance in PT between non-survivor and survivor cases, with a median (IQR) PT of 12.1 s (11.2–13.7) and 11.4 s (10.4–12.6), respectively (*P* ═ 0.004). The study also reported that elevated PT was associated with increased odds of in-hospital death [9].

A separate retrospective study, involving 380 patients, found that PT was significantly higher in critically ill patients than in those classified as severe or moderate [4]. The study also reported that PT increased with escalating severity of the disease (*P* < 0.0001). Furthermore, multivariable logistic regression analysis indicated that an elevated PT of more than 16 s was associated with an increased risk of death (OR ═ 4.94 [95% CI 1.50–16.25]; *P* ═ 0.001) [4]. Subsequent retrospective research revealed that individuals exhibiting D-dimer levels of ≥ 2.0 µg/mL had significantly elevated PT levels (*P* < 0.001) [79].

### Lactate dehydrogenase (LDH)

A previous study conducted in China, which included 191 patients, reported that the levels of LDH were higher in non-survivors than in survivors, with a median (IQR) LDH level of 521.0 U/L (363.0–669.0) and 253.5 U/L (219.0–318.0), respectively (*P* < 0.0001). Furthermore, elevated levels of LDH were associated with death [9].

According to another study conducted in China involving 1099 patients, out of which 173 were severe cases and 926 were non-severe cases, the reported LDH levels of ≥ 250 U/L were found in 72 out of 124 (58.1%) severe cases and 205 out of 551 (37.2%) non-severe cases [78]. Similarly, a study that included 380 patients with varying severity of disease showed that LDH levels were significantly different across all comparisons between critical, severe, and moderate COVID-19 patients (*P* < 0.0001) [4].

A recent study by Wu et al. reported that 135 of 198 patients (68.2%) had elevated LDH levels, with a median (IQR) LDH level of 307.50 U/L (232.25–389.25). Additionally, higher levels of LDH were associated with an increased risk of developing ARDS and death [11]. Another study evaluating predictors of respiratory failure showed that LDH levels at admission were significantly associated with respiratory failure [81].

### Troponin

According to four different studies conducted in Wuhan, China, elevated levels of troponin were observed in more severe or deceased patients [7, 9, 10, 80]. One of these studies revealed that patients with cardiac injury had higher levels of high-sensitivity troponin I than patients without cardiac injury, with a median (IQR) troponin level of 0.19 µg/L (0.08–1.12) and < 0.006 µg/L (< 0.006–0.009), respectively [10]. A second study reported that non-survivor patients had higher levels of high-sensitivity cardiac troponin I than the survivor group, with a median (IQR) troponin level of 22.2 pg/mL (5.6–83.1) and 3.0 pg/mL (1.1–5.5), respectively [9]. A third study reported that the levels of hypersensitive troponin I were higher in ICU patients than in non-ICU patients, with a median (IQR) troponin level of 11.0 pg/mL (5.6–26.4) and 5.1 pg/mL (2.1–9.8), respectively [7]. A fourth study found that cardiac troponin levels significantly differed between discharged and deceased patients [80]. Furthermore, the univariable analysis showed that elevated high-sensitivity cardiac troponin I was associated with death [9].

### Ferritin

In a study conducted by Liao et al. in Wuhan, China, it was shown that ferritin levels differed significantly between critical, severe, and moderate COVID-19 patients (*P* < 0.05). The median (IQR) levels of serum ferritin were 108.20 ng/mL (57.55–310.20) for moderate patients, 539.19 ng/mL (356.88–843.65) for severe patients, and 1146.20 ng/mL (733.80–1749.10) for critical patients [4]. Another study based in the same city found that serum ferritin levels were higher in non-survivors than in survivors, with a median (IQR) serum ferritin level of 1435.3 ng/mL (728.9–2000.0) for the non-survivor group and 503.2 ng/mL (264.0–921.5) for the survivor group. Furthermore, elevated ferritin levels were associated with an increased risk of in-hospital death [9].

A retrospective analysis based on a cohort of 201 individuals diagnosed with COVID-19-associated pneumonia, revealed that serum ferritin was among various factors linked to the development of ARDS. However, it was not found to be associated with mortality [11].

### White blood cells (WBC)

According to three different studies, elevated levels of WBC count were observed in severe or deceased cases of COVID-19. The first study, conducted in Wuhan, China, on a sample of 138 COVID-19 patients, showed that the WBC counts were higher in ICU patients than in non-ICU patients, with a median (IQR) WBC count of 6.6 × 10^9^/L (3.6–9.8) and 4.3 ×10^9^/L (3.3–5.4), respectively (*P* ═ 0.003). Additionally, the WBC count was higher in the non-survivor group than in the survivor group [7]. The second study, also conducted in Wuhan and involving 191 patients, showed that the WBC counts were higher in the non-survivors group than in the survivors group, with a median (IQR) WBC count of 9.8 × 10^9^/L (6.9–13.9) and 5.2 × 10^9^/L (4.3–7.7), respectively (*P* < 0.001) [9]. The third study performed a comparative analysis involving 416 patients, of whom 82 had a cardiac injury and 334 did not have a cardiac injury. The study found that the median leukocyte count was significantly higher in patients with cardiac injury (*P* < 0.001) [10].

Wu et al. [11] reported that approximately one-quarter of patients exhibited leukocytosis. Another study showed that the WBC levels were higher in ICU patients than in non-ICU patients, with a median (IQR) WBC count of 11.3 × 10^9^/L (5.8–12.1) and 5.7 × 10^9^/L (3.1–7.6), respectively (*P* ═ 0.011) [3]. Furthermore, data analysis of 150 patients with COVID-19 in Wuhan, China, showed significant differences in WBC counts between deceased and discharged patients [80].

### Neutrophil count

A recent study conducted in Wuhan, China, involving 138 COVID-19 patients found that the neutrophil counts were higher in ICU patients than in non-ICU patients, with a median (IQR) neutrophil count of 4.6 × 10^9^/L (2.6–7.9) and 2.7 × 10^9^/L (1.9–3.9), respectively (*P* < 0.001). Additionally, a dynamic profile of laboratory findings, tracked in a subgroup of 33 patients (5 non-survivors and 28 survivors) showed that neutrophil counts in the non-survivors continued to increase until death occurred [7]. Another study conducted at the Wuhan Asia General Hospital, which included 343 patients, revealed that patients with D-dimer levels of ≥ 2.0 µg/mL exhibited higher levels of neutrophils (*P* < 0.001) [79].

According to another study based in Wuhan, China, involving 201 patients diagnosed with COVID-19 pneumonia, about one-third (68 out of 197 [34.5%]) exhibited neutrophilia. Furthermore, the study identified neutrophilia as a risk factor for the development of ARDS and the subsequent progression from ARDS to death [11].

### Alanine transaminase (ALT)

A recent study conducted in Wuhan, China, involving 138 COVID-19, found that the ALT levels were higher in ICU patients compared to non-ICU patients, with a median (IQR) ALT level of 35 U/L (19–57) and 23 U/L (15–36), respectively (*P* ═ 0.007) [7]. Another study conducted in the same city, on a sample of 191 COVID-19 patients, found that the ALT levels were higher in non-survivors than in survivors, with a median (IQR) ALT level of 40.0 U/L (24.0–51.0) and 27.0 U/L (15.0–40.0), respectively (*P* ═ 0.005). Furthermore, univariable analysis showed that elevated ALT levels were associated with death [9].

According to another study conducted in Wuhan, China, involving 1099 patients, ALT levels were less commonly elevated upon admission. Specifically, 38/135 (28.1%) of severe cases and 120/606 (19.8%) of non-severe cases had ALT levels greater than 40 U/L [78]. Another study, which involved 201 patients with COVID-19-associated pneumonia, reported that 43 (21.7%) patients developed a liver injury, with a median (IQR) ALT level of 31.00 U/L (19.75–47.00) [11].

### Aspartate aminotransferase (AST)

A recent study involving 138 COVID-19 patients found that the AST levels were higher in ICU patients compared to non-ICU patients, with a median (IQR) AST level of 52 U/L (30–70) and 29 U/L (21–38), respectively (*P* < 0.001) [7]. Another study, conducted on a sample of 1099 patients, reported that AST levels were less commonly elevated upon admission. Specifically, 56/142 (39.4%) of severe patients and 112/615 (18.2%) of non-severe patients had AST levels greater than 40 U/L [78].

A study that compared cardiac injury to non-cardiac injury patients, found that the median (IQR) AST levels were 40 U/L (27–60) vs 29 U/L (21–40), respectively [10]. In a separate investigation encompassing a cohort of 194 individuals diagnosed with COVID-19-related pneumonia, the AST levels were significantly associated with the occurrence of ARDS. However, there was no significant correlation between AST levels and the progression from ARDS to death. Furthermore, among the 194 patients, 59 exhibited liver injury characterized by elevated AST levels, with a median (IQR) AST level of 33.00 U/L (26.00–45.00) [11].

### Creatine kinase (CK)

In a recent study involving 138 hospitalized patients with NCIP, the CK-myocardial band (CK–MB) levels were higher in ICU patients than in non-ICU patients, with a median (IQR) CK–MB level of 18 U/L (12–35) and 13 U/L (10–14), respectively (*P* < 0.001) [7].

Another study that compared patients with cardiac injury to those without cardiac injury, showed that the median (IQR) of CK–MB was significantly higher in the cardiac injury group (*P* < 0.001) [10]. In a separate study of 191 hospitalized patients with laboratory-confirmed COVID-19, the CK levels were higher in the non-survivors group consisting of 54 patients than in the survivors group consisting of 137 patients, with a median (IQR) of 39.0 U/L (19.5–151.0) against 18.0 U/L (12.5–52.1), respectively. Furthermore, a multivariable logistic regression analysis revealed that elevated CK levels were among a number of factors that exhibited a significant association with a higher likelihood of mortality [9].

### Creatinine

In a retrospective study involving 138 hospitalized patients with NCIP, the creatinine levels were higher in the ICU patients compared to the non-ICU patients, with a median (IQR) of 80 µmol/L (66–106) and 71 µmol/L (58–84), respectively (*P* ═ 0.04). Upon tracking the dynamic profile of 33 patients, of whom 5 deceased and 28 survived, it was observed that the creatinine levels continued to increase until death occurred [7]. Furthermore, data analysis of 150 COVID-19 patients in China showed significant differences in blood creatinine levels between the deceased and discharged patients [80].

A recent study involving 1099 patients reported that 6/138 (4.3%) severe patients and 6/614 (1.0%) non-severe patients had creatinine levels ≥ 133 µmol/L [78]. Another study of 191 hospitalized patients with laboratory-confirmed COVID-19 reported that creatinine was among several factors associated with death [9]. In contrast, Wu et al. [11] reported that while creatinine was one of several factors related to the development of ARDS, it was not associated with progression from ARDS to death.

A recent study indicated that the median (IQR) creatinine level was 11.5 mg/L (7.2–19.2) among patients with cardiac injury and 6.4 mg/L (5.4–7.8) among those without cardiac injury, which was a statistically significant difference (*P* < 0.001) [10]. Furthermore, Herold et al. [81] reported that creatinine levels at admission were significantly associated with respiratory failure.

### Procalcitonin

A previous study involving 191 hospitalized patients with laboratory-confirmed COVID-19 found that increased procalcitonin levels were linked to a higher likelihood of in-hospital mortality [9]. Another study of 1099 patients revealed that 16/117 (13.7%) severe cases and 19/516 (3.7%) non-severe cases had procalcitonin levels ≥ 0.5 ng/mL [78].

According to a study involving 41 COVID-19 patients, the majority of patients (69%) had normal procalcitonin levels upon admission (procalcitonin < 0.1 ng/m). Additionally, four ICU patients developed secondary infections, and three had procalcitonin levels greater than 0.5 ng/mL [3]. In another study involving 138 patients with NCIP, the laboratory findings indicated that 49 patients had procalcitonin levels ≥ 0.05 ng/mL. In addition, out of the total number of patients who had procalcitonin levels ≥ 0.05 ng/mL, 27 (75.0%) were from the ICU patients group and 22 (21.6%) were from the non-ICU patients group [7].

Based on a previous study involving 416 COVID-19 patients, the median (IQR) procalcitonin levels were 0.27 ng/mL (0.10–1.22) for those with cardiac injury vs 0.06 ng/mL (0.03–0.10) for those without cardiac injury. In addition, the overall study population showed elevated median (IQR) procalcitonin levels (0.07 ng/L [0.04–0.15]) [10].

### Blood urea nitrogen (BUN)

According to a retrospective study conducted in Wuhan, China, involving 138 hospitalized patients with NCIP, the BUN levels were higher in the ICU patients than in the non-ICU patients, with a median (IQR) BUN level of 5.9 mmol/L (4.3–9.6) and 4.0 mmol/L (3.1–5.1), respectively (*P* < 0.001) [7]. Additionally, data analysis of 150 COVID-19 patients in the same city showed significant differences in BUN levels between deceased and discharged patients [80].

### Lymphocyte counts

According to a retrospective study involving 138 hospitalized patients with NCIP [7], the lymphocyte counts were lower in the ICU patients than in the non-ICU patients, with a median (IQR) lymphocyte count of 0.8 × 10^9^/L (0.5–0.9) and 0.9 × 10^9^/L (0.6–1.2), respectively (*P* ═ 0.03). Upon tacking dynamic profiles of 33 patients, of whom 5 deceased and 28 survived, lymphocyte counts continued to decrease until death occurred [7]. Similarly, another study involving 41 patients with confirmed COVID-19 infection also indicated lymphopenia upon admission (lymphocyte count < 1.0 × 10^9^/L) in 26 (63%) patients [3].

Guan et al. reported that lymphocytopenia was present in 83.2% of patients upon admission. The prevalence of lymphocytopenia was higher in individuals with severe symptoms compared to those with non-severe symptoms. Furthermore, the median (IQR) lymphocyte counts were 800 per mm^3^ (600–1000) in severe patients and 1000 per mm^3^ (800–1400) in non-severe patients [78]. A different study of 150 COVID-19 patients showed significant differences in absolute lymphocyte values between deceased and discharged patients [80].

In a recent study involving 191 hospitalized patients diagnosed with COVID-19 through laboratory testing, lymphocyte counts were significantly lower in 54 patients who did not survive compared to 137 patients who did survive. The median (IQR) lymphocyte count in non-survivor cases was 0.6 × 10^9^/L (0.5–0.8), whereas in survivor cases it was 1.1 × 10^9^/L (0.8–1.5). Moreover, patients with lymphopenia exhibited an increased likelihood of experiencing in-hospital mortality [9]. In a study conducted by Liao et al. [4], it was demonstrated that an elevated neutrophil-to-lymphocyte ratio (≥ 9.13; OR ═ 5.39; *P* ═ 0.004) was correlated with increased mortality. Another study reported that lymphocyte counts were among several factors associated with the development of ARDS but not its progression to death. In addition, 64% out of 194 patients exhibited lymphocytopenia with a median (IQR) lymphocyte count of 0.91 × 10^9^/mL (0.60–1.29) [11].

In a previous study, the median (IQR) lymphocyte count was 600 cells/µL (400–900) in patients with cardiac injury vs 1000 cells/µL (800–1400) in patients without cardiac injury, with significant differences (*P* < 0.05). However, the median value of the lymphocyte count was within the normal range in the overall population analysis of 416 patients [10]. Another study conducted at Wuhan Asia General Hospital demonstrated that patients with D-dimer levels ≥ 2.0 mg/L had lower lymphocyte levels (*P* < 0.001) [79].

### Platelet counts

A recent study involving 1099 patients reported that thrombocytopenia was present in 36% of the patients. Additionally, the median (IQR) platelet counts were 137,500 per mm^3^ (99,000–179,500) in severe patients and 172,000 per mm^3^ (139,000–212,000) in non-severe patients [78]. Another study involving 150 COVID-19 patients revealed a significant difference (*P* < 0.05) in platelet counts between deceased and discharged patients [80].

Liao et al. analyzed a cohort of 380 hospitalized patients diagnosed with COVID-19 and revealed that critical patients exhibited significantly lower eosinophils and platelet levels compared to severe patients (*P* < 0.001). Furthermore, thrombocytopenia, defined as a platelet count below 100 × 10^9^/L, was observed in 42 out of 86 patients (49%) with critical disease. This rate was significantly higher compared to patients with severe disease, where thrombocytopenia was recorded in 14% of the patients, and moderate disease, where thrombocytopenia was recorded in 6% of the patients. Both of these differences were found to be statistically significant with *P* values less than 0.001. Furthermore, a multivariable logistic regression analysis revealed that thrombocytopenia was significantly associated with an elevated risk of mortality (OR ═ 8.33 [95% CI 2.56–27.15]; *P* < 0.001) [4].

In another study involving 191 hospitalized COVID-19 cases, the platelet counts were lower in 54 non-survivors compared to the 137 survivors, with a median (IQR) platelet count of 165.5 × 10^9^/L (107.0–229.0) against 220.0 × 10^9^/L (168.0–271.0) (*P* < 0.0001) [9]. A separate study conducted at Wuhan Asia General Hospital involving 343 patients showed that patients with D-dimer levels ≥ 2.0 mg/L had lower hemoglobin levels (*P* ═ 0.003) and platelet counts (*P* ═ 0.009) [79].

### Hemoglobin

A study involving 101 hospitalized COVID-19 patients found a statistically significant difference in hemoglobin levels across critical, severe, and moderate cases, with the lowest hemoglobin levels observed in critically ill patients [82]. In contrast, another study reported no significant difference in hemoglobin levels between survivor and non-survivor groups [9].

### Albumin

According to a study conducted in Korea involving 1952 COVID-19 patients, a statistically significant decrease in albumin levels was observed in patients with severe and moderate disease compared to those with mild disease (*P* < 0.001). Multinomial logistic regression analyses further revealed a significant association between abnormal albumin levels and the severity of the disease [83]. Similarly, another study reported that albumin levels were significantly lower in patients with critical disease compared to those with severe and moderate diseases [82].

In a separate study involving 191 COVID-19 patients, non-survivor patients had a lower albumin level than survivors (*P* < 0.0001) [9].

## Classification of COVID-19 severity

Based on the guidelines provided by the World Health Organization (WHO), COVID-19 is classified into three distinct levels of severity: non-severe (mild or moderate), severe, and critical. It is imperative to clearly differentiate patients classified as severe and those classified as non-severe to determine appropriate treatment strategies. Individuals afflicted with severe COVID-19 exhibit the manifestations of ARDS, sepsis, septic shock, organ dysfunction, or necessitate the administration of vasopressor therapy. They display symptoms of pneumonia and at least one of the following indicators: an SpO_2_ level below 93% while breathing room air, a respiratory rate exceeding 30 breaths per minute, experiencing severe respiratory distress, a ratio of the arterial partial pressure of oxygen (PaO2) to the fractional concentration of inspired oxygen (FiO2) equal to or less than 40 kPa, or an increase in lesion progression of over 50% within a 24 to 48-h period as observed in pulmonary imaging. Patients who do not show clinical manifestations indicative of severe or critical disease are categorized as non-severe cases [4, 5].

Individuals exhibiting mild symptoms, such as fever, malaise, cough, upper respiratory symptoms, and no indications of pneumonia on imaging, typically do not necessitate hospitalization. Those presenting with moderate symptoms may display signs like fever and respiratory distress, accompanied by radiographic evidence of pneumonia, but they do not show severe or critical manifestations.

Patients with critical disease are identified by respiratory failure that necessitates mechanical ventilation, shock, or other organ failures requiring ICU monitoring and treatment [4].

According to a report from the Chinese Center for Disease Control and Prevention (CDC), it was observed that, based on the examination of 72,314 patients, the majority of cases were categorized as mild. Specifically, 81% of these cases were identified as mild, either without pneumonia or presenting with mild pneumonia. Nevertheless, a notable proportion of cases, specifically 14%, exhibited severe symptoms. These symptoms included dyspnea, a respiratory frequency of 30 breaths per minute or more, blood oxygen saturation of 93% or lower, a PaO2 to FiO2 ratio below 300, and lung infiltrates expanding beyond 50% within 24–48 h. Moreover, 5% of the cases were classified as critical, with these patients suffering from respiratory failure, septic shock, and/or multiple organ dysfunction or failure [2].

In a separate study conducted in Jordan, researchers analyzed the hospitalization data of COVID-19 patients. Out of the total sample, it was found that 362 individuals, accounting for 39.0% of the sample, were categorized as non-severe cases. Additionally, 32.2% of the patients were classified as severe, while 28.7% were identified as critical. From this cohort, a majority of 77.3% were hospitalized and subsequently survived, while 22.7% did not survive their hospital stay [6].

## Comorbidities in COVID-19 patients

In a meta-analysis conducted on a sample size of 1786 patients, 1044 individuals (58.45%) were identified as male, and 742 individuals (41.55%) were identified as female. The participants had an average age of 41 years [84]. The most commonly reported comorbidities were hypertension (15.8%), cardiovascular and cerebrovascular disease (11.7%), and diabetes (9.4%) [9, 84]. Less commonly reported comorbidities included immunodeficiencies (0.01%), renal disorders (0.8%), respiratory illnesses (1.4%), malignancy (1.5%), HIV (1.5%), and hepatitis B (1.5%) [84]. Another study involving 340 hospitalized COVID-19 patients indicated that hypertension was the most common comorbidity, followed by diabetes, coronary heart disease, and carcinoma [4].

Clinical data from the COVID-19-Associated Hospitalization Surveillance Network (COVID-NET), which covered 1478 hospitalized COVID-19 patients, revealed that the leading comorbidity was hypertension (49.7%), closely followed by obesity (48.3%). Other reported comorbidities included chronic lung disease (34.6%), diabetes mellitus (28.3%), and cardiovascular disease (27.8%) [85]. Allwood et al. [86] further highlighted in their study that hypertension is the most prevalent comorbidity observed in ICU patients.

## Prevention of COVID-19

According to the CDC, several measures can help protect individuals from COVID-19 [87]. These measures include getting vaccinated, wearing a mask, and maintaining a distance of at least 6 ft from others. It is also recommended to avoid crowds and poorly ventilated spaces, regularly wash hands, cover coughs and sneezes, frequently clean and disinfect commonly touched objects and surfaces, monitor one’s health state daily, and undergo testing to prevent the spread of the virus to others [87].

## Inpatient management of COVID-19

Available trial data suggest that remdesivir may enhance the recovery rate in hospitalized patients with mild or moderate diseases who do not necessitate oxygen supplementation but are susceptible to progressing toward a more severe condition. However, the precise extent of its impact remains uncertain [88–90]. Another trial indicated no benefit from using dexamethasone in such patients, in fact, it may adversely affect their outcomes [91].

According to WHO guidelines, dexamethasone is recommended for patients diagnosed with severe or critical COVID-19. In instances where dexamethasone is unavailable, the use of other glucocorticoids in equivalent doses is considered reasonable [92]. The administration of IL-6 inhibitors, namely, tocilizumab and sarilumab, is strongly recommended for patients with severe or critical diseases [92]. This is because these inhibitors may prevent disease progression by blocking the inflammatory pathway [93]. The definitive efficacy of remdesivir is still under scrutiny. Some guideline panels, such as the WHO, recommend avoiding its administration in hospitalized patients due to the lack of definitive evidence supporting its benefits in enhancing outcomes in this population, such as reducing mortality rates or the requirement for mechanical ventilation [92].

## Complications of COVID-19

In a study conducted in Wuhan, China, involving 41 hospitalized COVID-19 patients, 100% of them had pneumonia, 29% developed ARDS, 15% suffered acute cardiac injury, and 12% experienced a secondary infection. Of the 41 patients, 68% were discharged, 10% were admitted to the ICU, and 15% died during hospitalization [3].

A recent study of 191 COVID-19 patients revealed that sepsis was the most commonly reported complication among patients, followed by respiratory failure, ARDS, heart failure, and septic shock. In addition, an analysis of 171 patients with complete data showed that the median (IQR) number of days from illness onset to complication occurrence was 9 days (7–13) for sepsis, 12 days (8–15) for ARDS, 15 days (10–17) for acute cardiac injury, 15 days (13–19) for acute kidney injury, and 17 days (13–19) for secondary infections [9]. Another study compared 84 patients who developed ARDS with 117 patients who did not develop ARDS [11]. The analysis showed that factors, such as comorbidities, lymphocyte counts, AST, prealbumin, creatinine, glucose, low-density lipoprotein, serum ferritin, and PT, were associated with ARDS development. However, these factors did not correlate with the incidence of death from ARDS [11].

In a study encompassing a cohort of 416 COVID-19 patients, it was observed that 82 patients exhibited cardiac injury, while 334 did not, 97 patients (23.3%) developed ARDS, while 8 (1.9%) developed acute kidney injury during their hospitalization [10]. Additional frequent complications observed in this study included electrolyte disturbances, hypoproteinemia, anemia, and coagulation disorders. These complications were reported in 30 patients (7.2%), 27 patients (6.5%), 13 patients (3.1%), and 12 patients (2.9%), respectively. Notably, it was observed that these complications were more prevalent in patients who experienced cardiac injury compared to those who did not, with the differences being statistically significant [10]. In another study encompassing 380 COVID-19 patients (out of which 86 were critical, 145 were severe, and 149 were moderate patients), hospital death occurred in 55 patients (14.47%) [4]. Among the non-survivors, ARDS was the most prevalent complication (69%), followed by septic shock (20%). Other observed complications included thrombotic and hemorrhagic events (35%), including DIC (15%), venous thromboembolism (5%), gastrointestinal bleeding (5%), acute myocardial infarction (4%), haematuresis (4%), and acute cerebral infarction (1%) [4].

In a study encompassing 1099 COVID-19 patients, 91.1% had pneumonia, 3.4% developed ARDS, and 1.1% experienced shock [78]. Furthermore, the incidence of pneumonia was higher in patients with severe diseases compared to those with non-severe diseases [78]. Similarly, another study reported that shock, ARDS, arrhythmia, and acute cardiac injury were common complications among 138 patients. Notably, the ICU patients were more susceptible to these complications than the non-ICU patients [7].

According to a report by the Chinese CDC covering 72,314 cases, the overall case-fatality rate (CFR) was 2.3% (representing 1023 deaths out of the 44,672 confirmed cases). There were no reported deaths among patients aged nine years or younger. In contrast, the CFR was 8.0% for those aged between 70 and 79, and 14.8% for those aged 80 or older. For patients with critical conditions, the CFR was 49.0%. Additionally, the CFR was higher among patients with pre-existing comorbidities, including cardiovascular diseases (10.5%), diabetes (7.3%), chronic respiratory diseases (6.3%), hypertension (6.05%), and cancer (5.6%) [2].

Some experts refer to the thromboinflammation observed in COVID-19 patients as COVID-19-associated coagulopathy (CAC) [27]. It appears to be distinct from DIC, although cases of DIC have been reported in severely affected COVID-19 patients.

A study evaluated 24 patients with severe COVID-19 pneumonia who were intubated. Alongside standard coagulation testing, they were assessed using other assays, such as VWF measurement and thromboelastography (TEG) [94]. Findings across various studies indicate a hypercoagulable state, characterized by extremely elevated levels of D-dimer, VWF antigen and activity, and factor VIII activity [28, 95]. TEG assessments on 44 ICU patients revealed a “fibrinolysis shutdown,” marked by a complete lack of clot lysis (lysis at 30 min [LY30] of 0%) in 57%. This condition was associated with a high incidence of kidney failure and thromboembolic events [96]. A separate study indicated that COVID-19 patients have higher platelet counts compared to patients with other coronavirus infections [97]. A case series from Ireland involving 50 patients in a regular medical ward reported similar findings as those seen in ICU patients, including elevated levels of D-dimer and fibrinogen, along with normal platelet counts and coagulation times [98]. Additionally, a separate study demonstrated that the ratio of VWF to a disintegrin and metalloproteinase with a thrombospondin type 1 motif, member 13 (ADAMTS13) enzyme varies depending on the disease severity [99].

An initial case series from Wuhan, China, which covered 183 consecutive patients, highlighted more pronounced thrombocytopenia and more significant prolongation of both PT and activated partial thromboplastin time (aPTT) [100–103]. The reasons for these differing findings, particularly when compared to later instances showing less severe PT and aPTT prolongation, remain uncertain. One potential estimation is that these patients might have been in a more critical condition, potentially due to the delayed disease recognition during the early stages of the pandemic. This could have led to delays in patient presentation and/or initiation of treatment.

In patients exhibiting prolonged aPTT, two studies detected a high rate of lupus anticoagulant, with rates of 88% and 91%, respectively [95, 104]. The lupus anticoagulant-positivity may correlate with thrombosis in COVID-19 patients [105]. While a lupus anticoagulant can result in an artifactual prolongation of the aPTT, it does not signify an increased risk of bleeding. Patients testing positive for the lupus anticoagulant should receive anticoagulation therapy if indicated.

There have been documented cases of immune thrombocytopenia (ITP) associated with COVID-19 [106–110]. Approximately 7% of critically ill patients exhibited a platelet count of 50,000/µL [110]. However, the underlying causes of thrombocytopenia were not explored in this study.

The hypercoagulable state associated with COVID-19 has, by some, been likened to a condition resembling DIC. This comparison is primarily due to the acute illness observed in many affected individuals, aligning them with the criteria for probable DIC set out by the 2009 scoring system of the International Society of Thrombosis and Haemostasis (ISTH) [111]. This ISTH scoring system, grounded in laboratory findings, is designed exclusively for patients with a confirmed underlying cause of DIC [111], which COVID-19 qualifies for due to its severity as an infection.

In COVID-19, the predominant clinical feature is thrombosis, in contrast to acute decompensated DIC, which is primarily characterized by bleeding symptoms. The laboratory indicators for COVID-19 differ from those of DIC. Although both conditions can manifest with elevated D-dimer levels, distinctive COVID-19 markers encompass heightened fibrinogen levels and increased factor VIII activity. This implies that coagulation factors are not extensively depleted in COVID-19 [94]. In one of the most extensive series focusing on thromboembolic events in COVID-19, none of the patients developed DIC [112].

Regardless of whether one emphasizes the differences or similarities to DIC, many of the fundamental principles of DIC management apply, including the importance of treating the underlying condition, basing interventions more on the clinical presentation rather than solely on laboratory testing, and providing anticoagulation for thrombosis alongside appropriate hemostatic therapies for bleeding.

Myocarditis is a rare side effect of the COVID-19 mRNA vaccines, particularly in young males. In a multi-center case series research, seven patients were diagnosed with myocarditis following the administration of BNT162b2 and mRNA-1273 COVID-19 vaccines. The findings from this study suggest a potential association between these vaccines and myocarditis [113]. Additionally, the literature reports 218 cases of COVID-19 vaccine-associated myocarditis [114]. In a recent systematic review, 396 published cases of myocarditis were reported, predominantly in male patients, following the administration of the second dose of the mRNA vaccine [115]. Notably, they experienced chest pain as a symptom.

Recent COVID-19 vaccinations, especially mRNA vaccines, have generated concerns for takotsubo (stress) cardiomyopathy. A recent systematic review encompassing ten studies and ten cases found that all patients exhibited high troponin levels and aberrant ECGs. In 90% of these patients, the left ventricular ejection fraction failed to reach 50% [116]. Thus, while rare, COVID-19 vaccination can lead to life-threatening takotsubo cardiomyopathy. Chest pain has been a concerning symptom for individuals after receiving both the first and second doses of the COVID-19 vaccine [116].

## Conclusion

Even though the COVID-19 pandemic has come to an end, the importance of understanding laboratory predictors for disease severity and mortality remains crucial. While the immediate crisis of the pandemic may have subsided, the knowledge gained from studying these predictors can have long-lasting implications. The insights gained from studying these predictors not only aid in identifying high-risk individuals but also facilitate targeted interventions, enabling healthcare systems to optimize resource allocation and deliver timely, appropriate care. Moreover, this knowledge significantly enhances our pandemic preparedness strategies, helping us to respond more swiftly and effectively to future infectious disease pandemics. Therefore, continued research into laboratory predictors for COVID-19 severity and mortality provides us with critical tools to safeguard public health and prevent potential future crises.
